# Dynamic infrared imaging of cerebrospinal fluid tracer influx into the brain

**DOI:** 10.1117/1.NPh.9.3.031915

**Published:** 2022-05-17

**Authors:** Samantha A. Keil, Molly Braun, Ryan O’Boyle, Mathew Sevao, Taylor Pedersen, Sanjana Agarwal, Deidre Jansson, Jeffrey J. Iliff

**Affiliations:** a VA Puget Sound Health Care System, VISN 20 Mental Illness Research, Education and Clinical Center (MIRECC), Seattle, Washington, United States; b University of Washington School of Medicine, Department of Psychiatry and Behavioral Sciences, Seattle, Washington, United States; c University of Washington School of Medicine, Department of Neurology, Seattle, Washington, United States

**Keywords:** glymphatic system, trans-cranial, dynamic, imaging, cerebrospinal fluid, interstitial fluid

## Abstract

**Significance:**

The glymphatic system has been described recently as a series of perivascular channels that facilitate fluid exchange and solute clearance in the brain. Glymphatic dysfunction has been implicated in numerous pathological conditions, including Alzheimer’s disease, traumatic brain injury, and stroke. Existing methods for assessing glymphatic function have been challenging: dynamic methods, such as two-photon microscopy and contrast-enhanced magnetic resonance imaging require expensive instrumentation and specific technical skills; slice-based fluorescent imaging is more readily implemented but lacks temporal resolution.

**Aim:**

To develop a straightforward and adaptable dynamic imaging approach for assessing glymphatic function *in vivo* in mice.

**Approach:**

Using a widely available small animal infrared (IR) imaging system (LICOR Pearl), visualization of IR cerebrospinal fluid tracer distribution over the cortical surface enables time-resolved measurement of the dynamics of glymphatic exchange. Using co-injection of IR and conventional fixable fluorescent tracers, dynamic imaging can be paired with whole-slice fluorescence imaging, permitting the quantification of glymphatic function throughout the brain as well as subsequent histological assessment.

**Results:**

These techniques were validated against one another, comparing differences between animals anesthetized with ketamine/xylazine and isoflurane.

**Conclusions:**

This technique permits sensitive dynamic imaging of glymphatic function, with the concurrent visualization of resolution of deeper structures.

## 1 Introduction

The glymphatic system, described over the last 10 years, supports the anatomically organized movement of fluid and clearance of solutes from the brain. Supported by astroglial aquaporin-4 water channels lining perivascular pathways, the glymphatic system facilitates cerebrospinal fluid (CSF) and interstitial fluid (ISF) exchange through the brain, removing interstitial solutes and waste products along peri-venous pathways.

Glymphatic exchange is most frequently assessed using slice-based fluorescent imaging after injection of fixable fluorescent tracer into the cisternal CSF.^1^^–^^6^ While this method allows for the assessment of tracer penetration and distribution with concomitant histological evaluation, it permits the assessment of CSF tracer distribution at only a single time point after injection. Thus, defining the time-course of CSF tracer influx requires many animals and much effort. In comparison, previous studies utilizing both two-photon^1^^,^^2^^,^^7^^,^^8^ and magnetic resonance imaging (MRI) methods^2^^,^^9^^–^^12^ have enabled *in vivo* imaging of tracer movement across time but have important limitations. Two-photon and MRI require expensive equipment and technical expertise that are not always readily available or affordable. In addition, two-photon imaging enables high-resolution imaging of tracer movement but can only image a very small field-of-view and often necessitates the use of invasive cranial windows that may affect normal glymphatic flux. MRI provides a wide field of view but cannot resolve down to microstructures. While these approaches provide sensitivity to time, they are limited by their anatomical scale and often require the access and use of expensive equipment.

Using the LICOR Pearl Trilogy small animal imaging system, we have developed a straightforward and novel dynamic imaging paradigm for visualization of infrared (IR) tracer distribution across the cortical surface in mice as a surrogate measure of glymphatic flux within the brain parenchyma. This approach is readily combined with fluorescence-based slice imaging to visualize tracer movement throughout brain tissue in addition to concurrent histological examination. This approach enables the combination of sensitive dynamic imaging over time and resolution of intraparenchymal structures that can be assessed in combination with histological targets of interest. Further, it uses affordable and widely available equipment, allowing for facile replication and widespread adoption of the technique.

## 2 Results

The purpose of this study was to develop and validate a novel dynamic imaging modality to quantify perivascular glymphatic exchange through the mouse brain in real time. A cocktail of two fluorescent tracers, including an inert IR carboxylate dye (IRDye 800CW, 1091 MW) and a fluorescently labeled dextran (Texas red dextran, 3000 MW), was injected into the cisterna magna, and tracer movement was imaged every 2 min from 20 to 40 min post-infusion using the LICOR Pearl Trilogy small animal imaging system. After *in vivo* imaging was complete, animals were perfusion-fixed, and IR and fluorescent surface imaging were performed on extracted whole brains. To validate this imaging technique against previously established slice-based methods of glymphatic imaging,^1^^–^^6^ fixed brains were processed, frozen, and cryosectioned, coronal slices were imaged, and fluorescence tracer distribution was quantified.

### 2.1 Dynamic Imaging of CSF IR Tracer Influx with the LICOR Pearl Imager

**Fig. 1:**
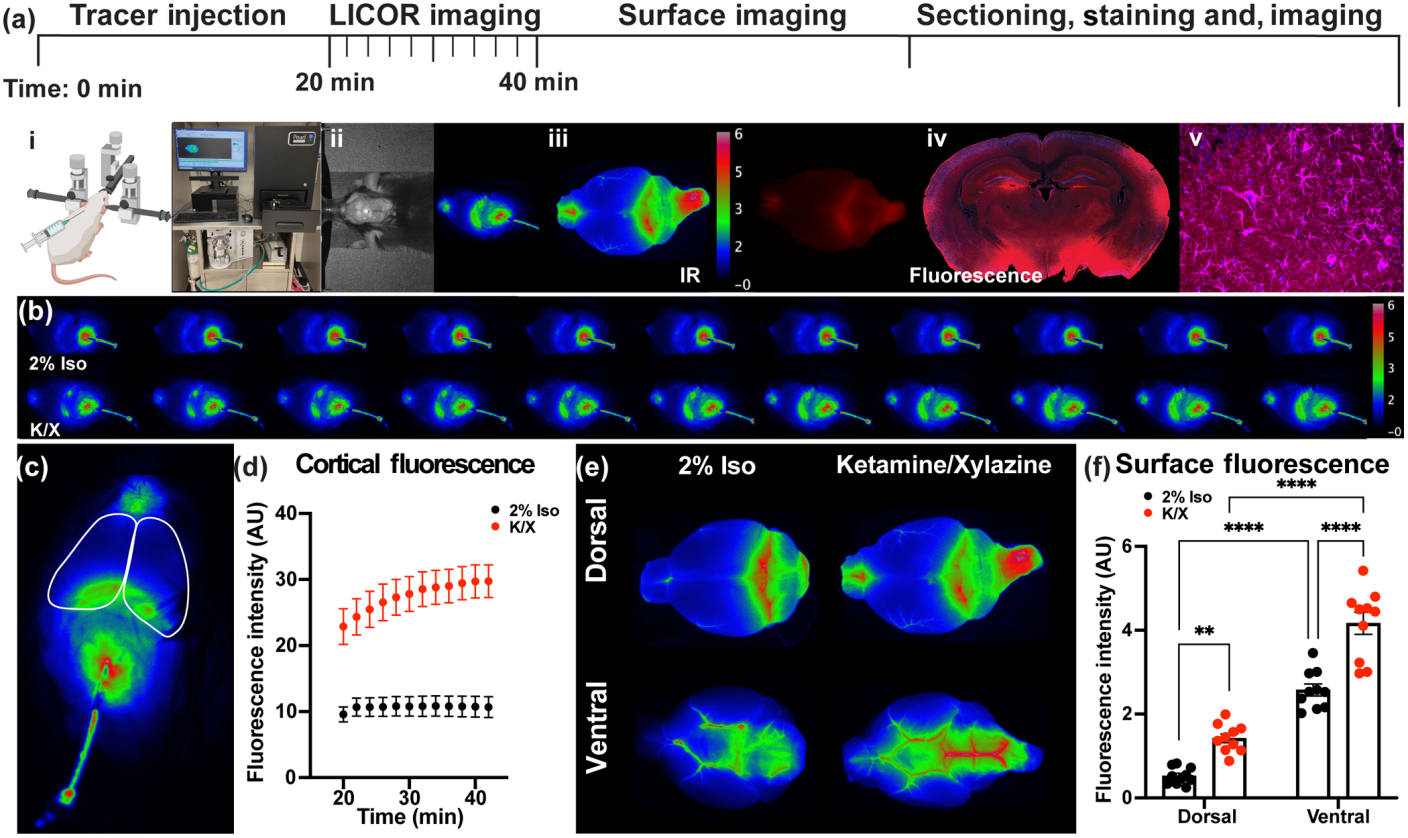
Dynamic IR imaging of CSF tracer influx. (a) Schematic of experimental time course with representative images showing injection of dual tracers into the cisterna magna at (i) t=0 and dynamic *in vivo* imaging of IR tracer movement with (ii) a LICOR Pearl Trilogy imaging system through an intact skull, followed by surface imaging of both (iii) IR and fluorescent tracer, and (iv) subsequent imaging of fluorescent tracer and (v) immunofluorescence-probed slices. (b) Representative dynamic time series of IR LICOR images from mice anesthetized with 2% Iso or K/X acquired every 2 min from 20 to 40-min post-tracer infusion. (c) Representative image of the left and right dorsal cortical surface ROIs used for analysis of dynamic transcranial images and (d) quantification of the average cortical fluorescent intensity of dynamic images over time (20 to 40 min). Data are mean ± SEM from n=10 mice/group and were analyzed by repeated measures two-way ANOVA followed by Šidák’s *post hoc* test (p<0.01 for time points 1–5, p<0.001 for time points 6–12). (e) Representative dorsal and ventral LICOR whole-brain surface images taken following perfusion and removal of the brain from the skull. (f) Comparative analysis of average fluorescence intensity of IR tracer of LICOR dorsal and ventral *ex vivo* surface images of mice anesthetized with K/X or 2% Iso (**p<0.0019; ****p <0.0001), data are mean ± SEM from n=10 mice/group and were analyzed by two-way ANOVA followed by Šidák’s *post hoc* test.

The primary goal was to determine if an IR CSF tracer could be dynamically imaged with the LICOR Pearl IR imaging system, allowing for assessment of tracer movement over the surface of the cerebral convexity as a surrogate measure of glymphatic exchange. The experimental pipeline [shown in Fig. 1(a)] describes the overall experimental approach including intracisternal injection of the tracers and dynamic imaging [Figs. 1(a.i) and 1(a.ii)], followed by *ex vivo* whole-brain surface imaging [Fig. 1(a.iii)] and tracer quantification in coronal brain slices [Fig. 1(a.iv)]. We also performed immunofluorescent co-staining and imaged with the fluorescent tracer, demonstrating the ability to follow the dynamic imaging of tracer influx with concomitant histological studies [Fig. 1(a.v)].

To determine whether this dynamic imaging technique could detect differences in perivascular glymphatic exchange, we evaluated well established effects of different anesthesia regimes on glymphatic function. Ketamine/xylazine (K/X) anesthesia modulates noradrenergic signaling and enhances glymphatic function,^5^^,^^6^^,^^12^ while isofluorane (Iso) impairs glymphatic exchange.^5^^,^^13^ Figure 1(b) and Fig. S1 in the Supplemental Material show representative imaging time series with dynamic tracer movement from t=20 to 40 min post-injection. Tracer accumulation was reflected by increased IR tracer intensity imaged over the dorsal brain surface. Cortical regions of interest (ROI) [Fig. 1(c)] were selected, and IR fluorescence intensity was quantified in mice under either Iso or K/X anesthesia [Fig. 1(d)]. Consistent with previous studies measuring tracer movement under Iso or K/X anesthesia,^2^^,^^3^^,^^5^^–^^7^^,^^14^ significantly higher IR tracer intensity was observed in K/X-treated animals across all timepoints compared to 2% Iso-treated animals [p<0.01, repeated measures two-way analysis of variance (ANOVA)]. *Ex vivo* brain surface images showed higher IR tracer intensity on the ventral surface compared to the dorsal surface in animals anesthetized with both Iso and K/X [Figs. 1(e) and 1(f)], which is consistent with anatomical movement of tracer after cisternal injection.^1^^,^^9^ Finally, both dorsal and ventral surface images show a significant increase in IR tracer distribution in K/X treated animals compared to 2% Iso treatment (p<0.01 and p<0.0001, repeated-measures two-way ANOVA, respectively).

### 2.2 Increased CSF Tracer Distribution in K/X-Treated Animals Assessed by Conventional Whole-Slice Fluorescence Imaging Following Dynamic IR Imaging

**Fig. 2:**
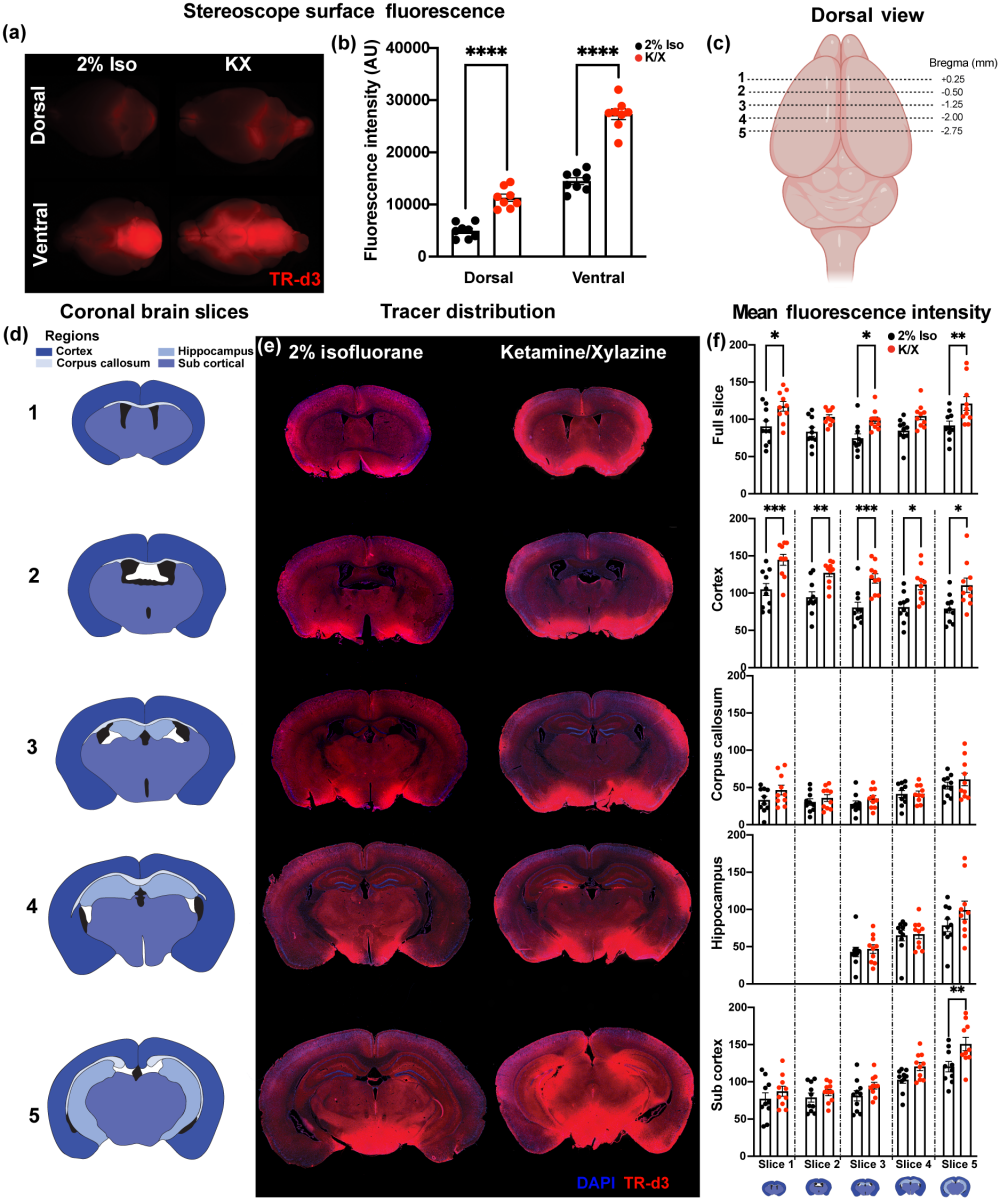
Fluorescence-based *ex vivo* imaging following dynamic IR imaging. (a) Stereoscopic fluorescence imaging of CSF tracer (Texas Red-conjugated 3 kD dextran, TR-d3) distribution over the dorsal and ventral brain surface. (b) Comparison of dorsal and ventral surface fluorescence in 2% Iso- or K/X-anesthetized animals. Data are mean ± SEM from n=8 mice/group and were analyzed by two-way ANOVA followed by Šidák’s *post hoc* test. (c) Schematic representation of cortical slice regions #1–5 moving anterior to posterior through the brain. Slice locations are shown as distance from bregma in mm. (d) Coronal ROI across five brain slices: cortex, corpus callosum, hippocampus, and subcortical regions. (e) Representative slice images #1–5 from a 2% Iso-anesthetized (left) and K/X-anesthetized animals (right). (f) Regional average fluorescence intensity of the full slice, cortex, corpus callosum, hippocampus, and subcortical regions between 2% Iso- and K/X-treated animals. Data are mean ± SEM from n=10 mice/group and were analyzed by two-way ANOVA followed by Šidák’s *post hoc* test (*p<0.05, **p<0.01, ***p<0.001, ****p<0.0001).

Next, we tested how these dynamic IR and surface imaging results compared to the method of slice-based quantification of fluorescence intensity, which has been a standard technique used to assess glymphatic function. A cocktail of IR carboxylate dye and fluorescent tracer was injected into the cisterna magna, allowing for direct comparison of fluorescence with IR imaging. Stereoscopic fluorescence imaging of the dorsal and ventral brain surfaces showed higher CSF tracer fluorescence on the ventral compared to the dorsal surface (p<0.0001), and a significant increase in fluorescence of brains from K/X-treated animals compared to 2% Iso-treated animals [Figs. 2(a) and 2(b), p<0.0001, repeated measures two-way ANOVA]. Five coronal brain slices (0.75-mm intervals anterior-posterior) were selected for analysis, and segmented into five ROIs [Figs. 2(c) and 2(d)]. Analysis of the mean fluorescence intensity across the entire brain slice showed that CSF tracer fluorescence was higher in K/X-treated animals compared to those treated with Iso [Figs. 2(e) and 2(f), p<0.0001 repeated-measures two-way ANOVA]. When regional analysis was conducted, CSF tracer fluorescence intensity was greater in the cortex (p<0.0001), corpus callosum (p=0.0378), and subcortical structures (p=0.0002) in K/X-treated animals compared to Iso-treated animals. *Post hoc* analysis revealed that the effect of K/X anesthesia on CSF tracer influx was distributed throughout the slices within the cortex, but differences in subcortical structures were driven by changes in the posterior brain. This *post hoc* analysis found no significant effect of anesthesia in the corpus callosum across slices. No significant effect of anesthesia was observed on CSF tracer influx into the hippocampus. As an alternative approach, we measured regional changes in threshold-based area coverage of CSF tracer fluorescence (Fig. S2 in the Supplemental Material). While this approach resulted in similar anesthesia-based differences in glymphatic influx, we observed that measurement of mean regional fluorescence resulted in less variable data.

### 2.3 Results from Dynamic IR and Whole-Slice Fluorescence Imaging Are Highly Correlated

**Fig. 3:**
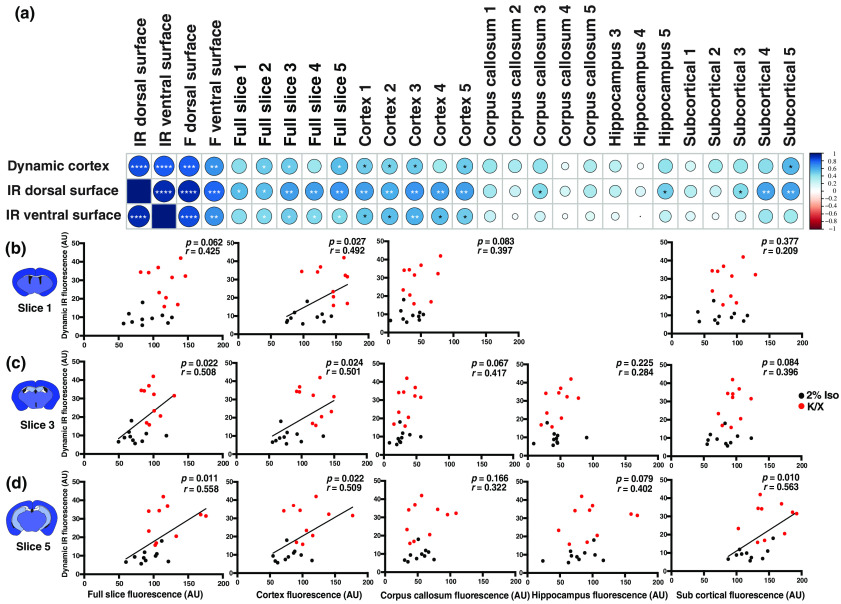
Association between *in vivo* dynamic IR imaging, *ex vivo* surface imaging, and fluorescent slice-based imaging. (a) Correlation table comparing *in vivo* dynamic IR tracer intensity (t=40  min) and *ex vivo* IR surface imaging with IR and fluorescence (F) surface imaging, and slice-based fluorescence imaging across the full slice or specific ROI. (*p<0.05, **p<0.01, ***p<0.001, ****p<0.0001; Pearson’s correlation coefficient reflected in heatmap). (b)–(d) Pearson correlation analysis between the *in vivo* dynamic LICOR IR intensity (t=40  min) and coronal slice fluorescence intensity of the full slice as well as the cortex, corpus callosum, hippocampus, and subcortical regions within slices 1, 3, and 5, respectively (Pearson’s correlation, r, and p values as noted).

The effect of anesthesia on glymphatic function assessed by dynamic IR imaging was consistent with prior studies reporting increased glymphatic function in K/X-versus Iso-treated animals.^5^^,^^15^ Yet it is unclear whether the results of dynamic IR imaging through the skull and over the dorsal brain surface are consistent with the results gained through more conventional slice-based fluorescence imaging. We compared IR and fluorescence intensity values from the final dynamic image taken at 40-min post-injection to the fluorescent intensity of the surface and slice images. Dynamic image fluorescence significantly correlates with IR dorsal and ventral surface images, fluorescent dorsal and ventral surface images, and full slice and region-specific slice fluorescence [Fig. 3(a)]. Plotting individual values of dynamic IR intensity against slice intensity from slices 1, 3, and 5 reveals a significant correlation between IR intensity and full slice fluorescence and cortical regions in slices 1, 3, and 5 [Figs. 3(b)–3(d)]. No significant correlations are seen between the dynamic IR image fluorescence and hippocampal or white matter ROIs, and among the subcortical ROIs, a significant association was observed only posteriorly.

### 2.4 Concurrent Assessment of Histological Immunofluorescence with Dynamic IR Imaging

**Fig. 4:**
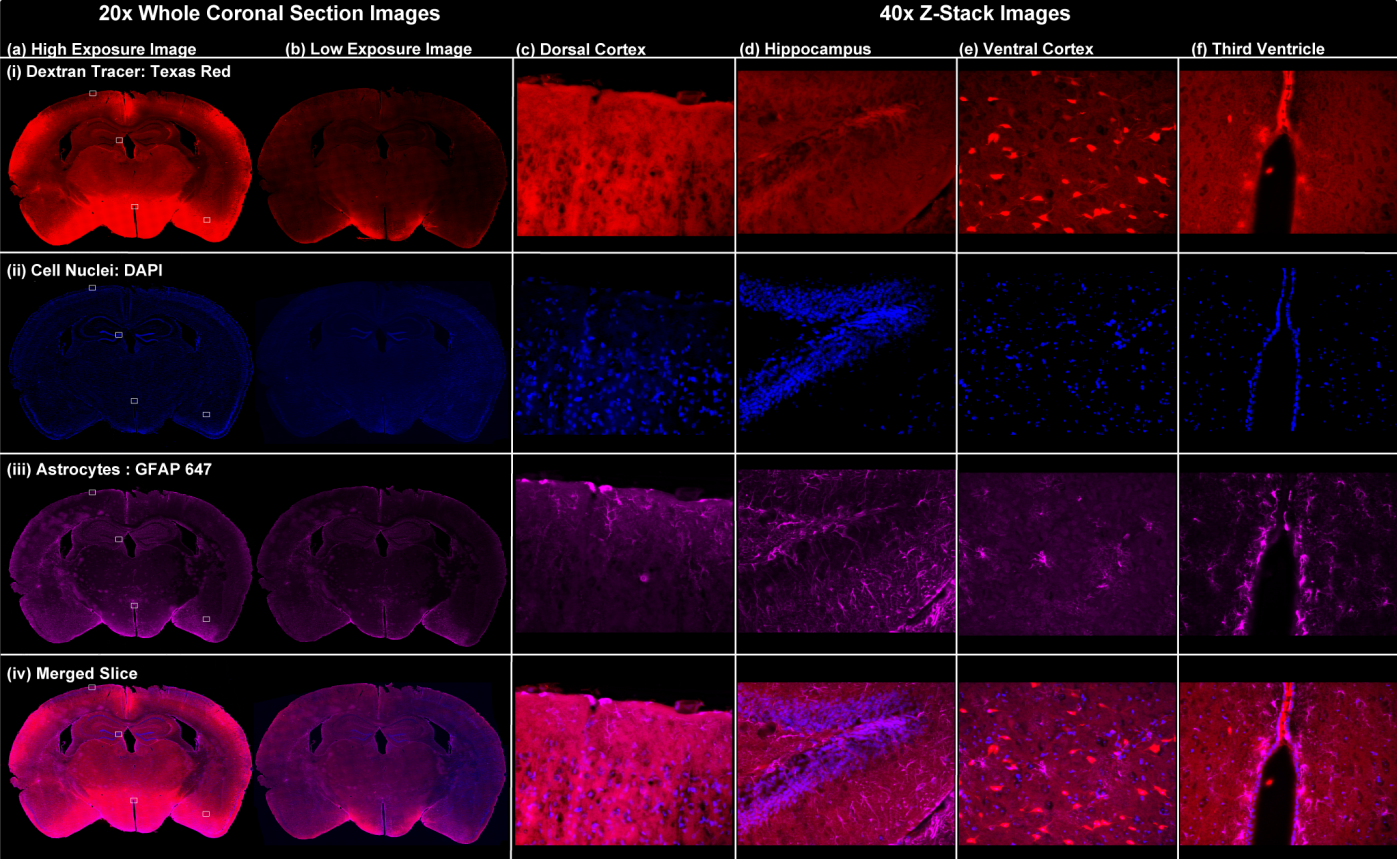
Assessment of histological outcomes after dynamic IR imaging. At left, representative stitched 20× coronal slice images from a mouse anesthetized with K/X and injected intracisternally with both IR and fluorescence CSF tracer, perfusion-fixed 45-min post-injection, and immunostained for the astroglial marker GFAP. Whole-slice images were acquired at (a) higher and (b) lower exposure times to capture the wide range in fluorescence intensity observed throughout the slice. At right, representative 40× Z-stack images from the (c) dorsal cortex, (d) hippocampus, (e) ventral cortex, and (f) third ventricle.

We next demonstrated the compatibility of dynamic IR imaging of CSF tracer influx using the LICOR Pearl system, slice-based fluorescence imaging of CSF tracer influx, and concurrent histological examination. As shown in Fig. 4, a representative 20-μm coronal brain slice from a K/X-anesthetized animal probed for the astroglial marker glial fibrillary acidic protein (GFAP) by immunofluorescence. Both longer- and shorter-exposure time 20× stitched images are provided. Higher magnification (40×) zoomed images are also provided showing CSF tracer distribution, GFAP immunofluorescence, and 4′,6-diamidino-2-phenylindole (DAPI) counter-labeling across distinct brain regions.

## 3 Discussion

We have developed a straightforward and novel experimental paradigm utilizing the LICOR Pearl Trilogy small animal imaging system to address the need for combined dynamic imaging and histologic assessment in glymphatic research. This technique offers an opportunity for dynamic imaging of IR tracer distribution across the cortical surface with the ability to follow up with gold standard slice-based fluorescence analysis and histological evaluation.

Specifically, this study evaluated CSF tracer distribution through dynamic and slice-based imaging of an IR and fluorescent tracer cocktail injected directly into the cisterna magna. The IR tracer distribution was monitored over 20- to 40-min post-injection in animals anesthetized with either K/X or Iso, after which animals were perfusion-fixed and whole-slice fluorescence imaging was conducted. Consistent with prior studies reporting enhanced glymphatic exchange with the use of K/X anesthesia versus suppression of perivascular exchange in animals under Iso anesthesia,^5^^,^^6^^,^^13^^,^^15^^–^^19^ we observed higher CSF tracer influx in K/X-anesthetized animals using dynamic IR imaging, IR imaging of tracer distribution of the fixed brain surface, fluorescence imaging of tracer distribution over the brain surface, and slice-based fluorescence imaging. This provides an important positive control, validating that dynamic IR imaging can capture physiological differences in glymphatic function induced by anesthetic state.

Dynamic IR imaging of CSF tracer distribution over and across the cortical surface, and IR imaging of the fixed dorsal and ventral brain surfaces may not faithfully reflect CSF tracer distribution into deeper brain regions. Thus, we conducted a correlation analysis between dynamic cortical IR intensity at 40-min post-injection, ventral and dorsal brain surface IR intensity, and regional CSF tracer distribution captured by conventional whole-slice fluorescence imaging. For these analyses, we pooled data from both K/X- and Iso-anesthetized animals. As expected, we observed that dynamic IR, fixed dorsal surface IR, and ventral surface IR intensity were strongly inter-correlated. These measures were significantly correlated with whole-slice fluorescence, and with cortical fluorescence across the rostro-caudal axis. Dynamic IR and dorsal surface IR were generally not associated with white matter, hippocampal, or subcortical gray matter measures of CSF tracer influx. Ventral surface IR was additionally associated with subcortical CSF tracer fluorescence, but mainly in posterior segments. These data place two key limitations on such trans-cranial and whole-slice imaging approaches. First, trans-cranial glymphatic imaging approaches faithfully reflect CSF tracer distribution into and through the cortical mantle, but do not reflect glymphatic exchange into subcortical structures. Second, whole-slice fluorescence—a commonly used measure of glymphatic function—may be dominated by changes in cortical glymphatic function and may not reflect exchange that occurs throughout different brain regions.

This approach permits the facile assessment of glymphatic function, allowing both dynamic and structural insights to be assessed in parallel. Like two-photon microscopy and dynamic contrast-enhanced MRI, dynamic IR tracer imaging provides the temporal resolution necessary to evaluate glymphatic flux within the brain but without the need for expensive equipment and complex technical expertise. The dynamic IR imaging and the *ex vivo* surface images are comparable, and in some ways potentially superior, when evaluating cortical tracer influx. In cases where superficial cortical fluid changes are the outcome of interest, and the study does not require the high spatial resolution of two-photon microscopy, this dynamic IR imaging technology might be considered as an alternative to these prior methods. While in principle a similar approach to IR imaging might be feasible in rats, including perhaps through a thinned skull preparation, the LICOR Pearl imaging system that we utilized is suitable only for imaging animals similar in size to mice. Thus one limitation to the present approach would be its limitation to use with mice.

As the role of the glymphatic system in cranial fluid dynamics and waste clearance from the brain has become more apparent,^1^^,^^6^^,^^7^^,^^9^^,^^20^ the ability to capture spatial and anatomical changes throughout the brain provides valuable insight. Here we have shown that *in vivo* dynamic IR imaging can be immediately followed by *ex vivo* imaging techniques and histologic evaluation to add further comprehensive spatial analysis in addition to temporal resolution. Our data show that this IR tracer fluorescence is most reflective of cortical CSF tracer influx, while a more informative regional assessment is possible through a slice-based approach. The ability to perform *ex vivo* experiments would also allow for follow-up staining and analysis of regional accumulation of pathologic proteins or other targets of interest in relation to tracer influx.

While previous research has implicated glymphatic system impairment in the development and progression of many neuropathologic disease states,^7^^,^^21^^–^^35^ the anatomically distributed nature of this system and relative optical inaccessibility necessitates the development of approachable and reproducible experimental paradigms enabling comprehensive progress within the field. Here, we have developed a dual IR and fluorescence imaging technique that when combined can analyze both temporal and spatial glymphatic dynamics. In the future, this technique could be used to further explore glymphatic clearance across neuropathologic disease states.

## 4 Materials and Methods

### 4.1 Mice

Wild type C57BL/6 male mice aged 11 weeks were maintained on a 12-h light/12-h dark cycle with food and water ad libitum. All experiments were carried out following the National Institutes of Health (NIH) Guide for Care and Use of Laboratory Animals and approved by the Institutional Animal Care and Use Committee of the University of Washington, Seattle.

### 4.2 Intracisternal Tracer Injection

Dual IR (non-fixable) and paraformaldehyde (PFA) fixable fluorescent CSF tracer was prepared with Texas red-dextran (3 kD, TR-d3; Invitrogen) at a concentration of 0.5% and 100  μM IR dye (LI-COR 800CW carboxylate 100 nM; Fisher Scientific) in artificial CSF. Mice were anesthetized with either 2% Iso or a K/X cocktail (intraperitoneal; 130-mg/kg ketamine, 8.8-mg/kg xylazine, and 0.02-ml/g body weight). Once anesthetized, mice were fixed in a stereotaxic frame, the dura mater was exposed through blunt dissection of neck muscles below the occipital crest, and a 30G steel needle was inserted at 45 deg (relative to the mouse head) 1 to 2 mm into the cisterna magna.^36^ Once the cannula was secured to the dural membrane with cyanoacrylate glue, 10  μl of CSF tracer was injected over a 10-min period with a syringe pump (Harvard Apparatus). Following the 10-min injection period, CSF tracer distribution was permitted for an additional 5 min.

### 4.3 LICOR Dynamic Imaging

To visualize tracer movement from the subarachnoid space of the cisterna magna into the brain parenchyma, mice were placed on the heated LICOR Pearl imager platform. Animals in the 2% Iso anesthesia treatment group were maintained under 2% Iso, while K/X treated animals received an additional 1/3 dose of K/X by weight 45 min after the first K/X dose to maintain the plane of anesthesia. Transcranial images were taken of the exposed skull surface every 2 min from 20 min post tracer injection to 40 min post tracer injection. Imaging from 20 to 40 min captures tracer influx into the mouse cortex, however, longer imaging timepoints could be used depending on the outcome of interest. In the present study, we sought to conduct perfusion fixation at 45-min post-injection, thus dynamic imaging ended at t=40  min post-injection. Imaging was performed at 85  μm/pixel, though images can also be acquired at 170 or 255  μm/pixel.

### 4.4 Perfusion and Sectioning

After 40 min of tracer influx and imaging, mice were transcardially perfused with ice-cold phosphate-buffered saline (PBS), followed by 4% PFA. Brains were removed, and images of the dorsal and ventral surfaces of the extracted brain were taken on the LICOR. Brains were then post-fixed overnight in 4% PFA at 4°C, protected from light. Brains were rinsed in PBS and cryoprotected in 30% sucrose until saturated.

### 4.5 *Ex Vivo* Fluorescence Imaging

Once brains are appropriately cryoprotected in 30% sucrose, dorsal and ventral surface fluorescent and bright-field images were acquired using fluorescent stereoscope images. Brains were then fixed in optical coherence tomography and maintained at −80°C and then cryosectioned in 20-μm coronal sections and mounted with ProLong™ Diamond Antifade Mountant with DAPI. Fluorescent penetration from the cisterna magna was imaged on five *ex vivo* sections rostral to caudal through the brain with a Keyence BZ-X800 fluorescence scope. Tile scans were taken at 10× magnification, and images were stitched and saved as TIFF files for analysis.

### 4.6 Immunofluorescence

Additional representative immunofluorescence was performed on frozen K/X treated 20-μm slices. Slices were PBS washed and blocked for 1 h at room temperature with 2% BSA in PBS-Triton (0.3%) with 5% donkey serum, incubated with primary antibody overnight at 4°C, and incubated with secondary antibody for 2 h at room temperature. Primary and secondary antibodies used for representative staining were rabbit anti-GFAP (1:750, Abcam) and donkey anti-rabbit Alexa Fluor 647 (1:500; Life Technologies).

Once antibody staining was complete, the section was mounted as described above and imaged at 20× magnification with the Keyence microscope. Representative 40× images were also taken within the left hippocampus.

### 4.7 Image Processing and Analysis

Stitched fluorescent images were processed in ImageJ. Overlay images were RGB separated, and the DAPI/blue channel was used to manually draw ROI for the cortex, corpus callosum, hippocampus, and sub-cortical regions as shown in Fig. 2(d). These regions were used to calculate the mean raw fluorescence intensity for each region within that section. Additional analysis of slice 4 was performed with manual thresholding to remove background fluorescence to confirm that approaches utilizing raw fluorescence intensity and threshold-based assessment are comparable. This is visually represented in Fig. S2 in the Supplemental Material.

### 4.8 Statistical Analysis

All statistical testing was performed on GraphPad Prism 9.2 and was assembled in Adobe Illustrator. Tests were chosen based on the data set being analyzed and are reported in the figure legends. They include repeated-measures two-way ANOVA, Sidak’s multiple comparisons test, and Pearson’s correlation. All statistical testing was two tailed, and exact p values were calculated at a 0.05 level of significance. All values are expressed as the mean ± standard error of the mean (SEM).
