# Supplementary material for: Dynamic infrared imaging of cerebrospinal fluid tracer influx into the brain
Source: Neurophotonics. 2022 May 17;9(3):031915. doi: 10.1117/1.NPh.9.3.031915 (PMC9113559; doi:10.1117/1.NPh.9.3.031915)
Supplement: Supplementary file 1 [file NPh_009_031915_SD001.docx]

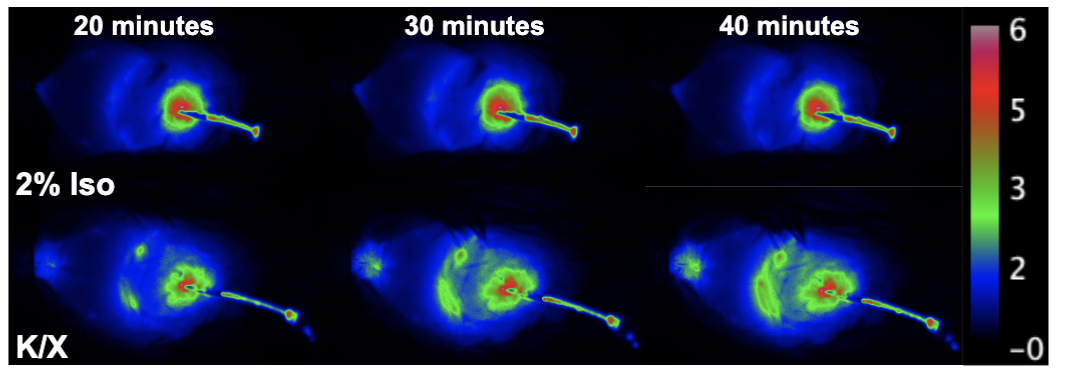
**Supplemental Figure 1.** Representative dynamic IR LICOR images from mice anesthetized with 2% isofluorane (Iso) or ketamine/xylazine (K/X). Shown here are representative images taken at 20, 30, and 40 minutes for each animal.

**
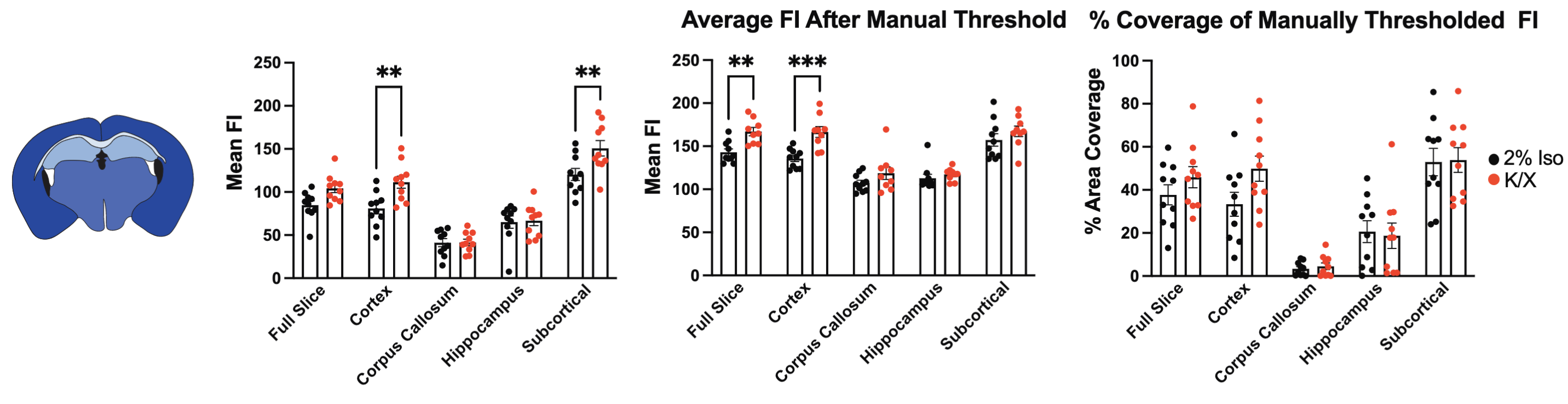
**

**Supplemental Figure 2.** Comparison of raw fluorescence intensity to threshold-based area coverage assessment. Comparative analysis of slice 4 as shown in representative schematic (at left). Values resulting from measurement of mean fluorescence intensity (middle) and threshold-based assessment of CSF tracer area coverage (right) show similar results when comparing the effects of Iso- and K/X anesthesia on glymphatic function.
